# Observational study: 27 years of severe malaria surveillance in Kilifi, Kenya

**DOI:** 10.1186/s12916-019-1359-9

**Published:** 2019-07-08

**Authors:** Patricia Njuguna, Kathryn Maitland, Amek Nyaguara, Daniel Mwanga, Polycarp Mogeni, Neema Mturi, Shebe Mohammed, Gabriel Mwambingu, Caroline Ngetsa, Kenedy Awuondo, Brett Lowe, Ifedayo Adetifa, J. Anthony G. Scott, Thomas N. Williams, Sarah Atkinson, Faith Osier, Robert W. Snow, Kevin Marsh, Benjamin Tsofa, Norbert Peshu, Mainga Hamaluba, James A. Berkley, Charles R. J. Newton, John Fondo, Anisa Omar, Philip Bejon

**Affiliations:** 1 KEMRI-Wellcome Trust Research Programme, CGMR-C, KEMRI, PO Box 230, Kilifi, Kenya; 2 Department of Paediatrics, Faculty of Medicine, Imperial College, London, UK; 3 Centre for Tropical Medicine and Global Health, Nuffield Department of Medicine, University of Oxford, Oxford, UK; 4 London School of Hygiene and Tropical Medicine, London, UK; 5 Department of Paediatrics, University of Oxford, Oxford, UK; 6 Department of Psychiatry, University of Oxford, Oxford, UK; 7 Kilifi County Department of Health, Kilifi, Kenya

**Keywords:** Severe malaria, Secular trend, Mortality, Africa, Longitudinal surveillance

## Abstract

**Background:**

Many parts of Africa have witnessed reductions in *Plasmodium falciparum* transmission over the last 15 years. Since immunity to malaria is acquired more rapidly at higher transmission, the slower acquisition of immunity at lower transmission may partially offset the benefits of reductions in transmission. We examined the clinical spectrum of disease and predictors of mortality after sustained changes in transmission intensity, using data collected from 1989 to 2016.

**Methods:**

We conducted a temporal observational analysis of 18,000 children, aged 14 days to 14 years old, who were admitted to Kilifi County Hospital, Kenya, from 1989 to 2016 with malaria. We describe the trends over time of the clinical and laboratory criteria for severe malaria and associated risk of mortality.

**Results:**

During the time periods 1989–2003, 2004–2008, and 2009–2016, Kilifi County Hospital admitted averages of 657, 310, and 174 cases of severe malaria per year including averages of 48, 14, and 12 malaria-associated deaths per year, respectively. The median ages in years of children admitted with cerebral malaria, severe anaemia, and malaria-associated mortality were 3.0 (95% confidence interval (CI) 2.2–3.9), 1.1 (95% CI 0.9–1.4), and 1.1 (95% CI 0.3–2.2) in the year 1989, rising to 4.9 (95% CI 3.9–5.9), 3.8 (95% CI 2.5–7.1), and 5 (95% CI 3.3–6.3) in the year 2016. The ratio of children with cerebral malaria to severe anaemia rose from 1:2 before 2004 to 3:2 after 2009. Hyperparasitaemia was a risk factor for death after 2009 but not in earlier time periods.

**Conclusion:**

Despite the evidence of slower acquisition of immunity, continued reductions in the numbers of cases of severe malaria resulted in lower overall mortality. Our temporal data are limited to a single site, albeit potentially applicable to a secular trend present in many parts of Africa.

**Electronic supplementary material:**

The online version of this article (10.1186/s12916-019-1359-9) contains supplementary material, which is available to authorized users.

## Background

*Plasmodium falciparum* malaria is an important cause of childhood morbidity and mortality in sub-Saharan Africa (sSA), which accounts for 90% of the world’s malaria deaths [1]. Most malaria episodes are successfully treated without hospital admission, but a small proportion of children are admitted to hospital for severe complications which may include coma (i.e. cerebral malaria), severe anaemia (necessitating urgent blood transfusion) or deep breathing (due to severe metabolic acidosis) [2, 3]. Globally, unprecedented reductions in *Plasmodium falciparum* transmission were observed from 2000 to 2010, but progress has stalled since 2010 [4] and the global mortality from malaria may now be increasing [1].

Immunity to clinical malaria is acquired following repeated exposure to malaria parasites. In high transmission areas, significant clinical malaria is rare after 5 years of age, owing to the acquisition of immunity in early childhood. By contrast, in low transmission areas, clinical malaria occurs among older children [5].

The dominant severe malaria phenotype in areas of low transmission is cerebral malaria rather than severe anaemia [5]. Cerebral malaria is associated with higher case fatality than severe anaemia, even when the most effective anti-malarial treatment artesunate is used [6, 7], and in addition, case fatality may be higher at older age [8]. As many parts of sSA approach lower transmission intensities, the changing clinical spectrum of severe malaria could potentially offset some of the gains in mortality that would otherwise be expected by improved malaria control [9].

A decline in transmission occurred between 2000 and 2010 in many parts of Africa including the East African Coast [4]. Detailed analysis of community parasite prevalence surveys on the Kenyan Coast show that the most recent decline began in the mid-1990s and that transmission increased again after 2010 [10]. In previous work, we found similar trends confirmed using yearly parasite prevalences from children admitted to hospital with trauma [11].

We previously reported reduced mortality and morbidity during the period of declining malaria transmission in Kilifi County Hospital (KCH) in Coastal Kenya from 1989 to 2008 [12]. However, transmission has increased since 2008, and we showed increasing rates of malaria among older children admitted to hospital between 2009 and 2014 [11]. We now report outcomes to 2016 including detailed clinical data to examine the impact that reduced immunity might have on clinical features and case fatality.

## Methods

Since May 1989, there has been continuous surveillance of hospital admissions to KCH as a partnership between the KEMRI-Wellcome Trust Research Programme and Kilifi County Department of Health. Consent for the use of data is sought from the parents or legal guardians of admitted children, and wider community engagement to explain research activity was undertaken [13]. Linkage to the Kilifi Health and Demographic Surveillance System (KHDSS) was established in 2002 [14]. At the midpoint of the surveillance period, the KHDSS comprised 250,000 residents, including 46,000 children below 5 years of age and 110,000 children below 14 years of age. Malaria control activities include distributions of insecticide-treated bed nets [15], but no indoor residual spraying. The malaria vaccine RTS,S was tested in clinical trials but has not been used routinely [16, 17].

The KHDSS covers an 891 km^2^ area, which included 202,000 individuals and 104,000 < 14-year-old children in 2002, rising to 287,000 individuals and 134,000 < 14-year-old children in 2016. The average age among the population of children below 14 years of age rose marginally from 6.7 years in 2002 to 7.0 years in 2016. No shifts in reported ethnicity are noted since 2002 to date. There are 27 major government primary health care facilities in the area in 2016, and in the absence of prospective monitoring data, we estimate that 7 of these are newly constructed in the last 15 years. User fees for healthcare access were introduced in Kenya in 1989 and removed in 2004, albeit with variable implementation and ongoing charges for inpatient care [18]. There have been no changes in the admission criteria in Kilifi during the study. We note reports of increasing urbanization in Kilifi [19]. The asymptomatic parasite prevalences from children admitted with acute trauma are given in Additional file 1: Table S1, illustrating the decline in transmission during the study period.

### Clinical surveillance

The pediatric service at KCH includes two wards; a 70-bed general ward and a 15-bed high dependency unit (HDU) staffed by research clinicians and nurses. The HDU admits children with serious illness requiring more intensive monitoring and management (albeit without mechanical ventilation facilities). Structured case records are completed electronically on all admissions, capturing age, residence, vital signs, clinical history and examination, and the Blantyre coma score [20]. Data are linked to the KHDSS database. All admissions routinely have a malaria blood slide, full blood count, blood glucose, and blood culture investigations. An extended biochemical screen, including blood gases, is included for children with severe illness. Ward clinicians review the hospital notes on discharge and assign one or two diagnostic terms. Inpatient treatment for malaria was parenteral quinine between 1989 and 2010. In 2010, parenteral treatment was changed to artesunate [6]. When children were able to take oral medication, this was chloroquine until 1998, then sulfadoxine/ pyramethamine until 2003, amodiaquine until 2005, and then artemether/lumefantrine to date.

### Definitions

In defining severe *P. falciparum* malaria, we used the clinical criteria specified by the WHO [21] (i.e. any one of the following: (a) cerebral malaria (defined as Blantyre coma score of < 3), (b) severe malaria anaemia (defined as hemoglobin concentration less than 5 g/dL), (c) respiratory distress (defined as deep breathing as assessed by a clinician), (d) prostration (defined as inability to stand in children who can usually stand, inability to sit in children who cannot usually stand but can sit, or inability to breastfeed in children who cannot usually sit or stand), (e) multiple convulsions (defined as two or more convulsions in the 24-h period prior to admission), (f) jaundice (defined clinically), (g) compensated shock (defined by age-specific increases in heart rate (in beats per minute (bpm) > 180 for children < 12 months of age; > 160 bpm for 12 months to 5 years of age; and > 140 bpm for children > 5 years of age) plus a capillary refill time of > 2 s), (h) decompensated shock (defined as systolic blood pressure < 50 mmHg), (i) kidney injury (defined according to age-specific pRIFLE criteria to calculate estimated glomerular filtration rates [22], using actual height and weight where available, and imputing an age-specific average height and weight for where these were not measured), (j) hypoglycaemia (defined as glucose < 2.2 mmol/l in accordance with WHO guidelines, although a threshold of 3 mmol/l was used for clinical management), (k) hyperparasitaemia (defined as parasite density > 250,000 parasites per μl)).

In addition to the WHO criteria, we added a parasite threshold to improve specificity. Asymptomatic parasitaemia is frequent in malaria-endemic regions, and clinical features of severe malaria overlap with other common causes of admission. We countered this by restricting analysis to parasitaemia above 2500 parasites/μl [23]. As a secondary sensitivity analysis, we re-ran all analyses including malaria parasitaemia at any density, with the additional restriction to children where “malaria” was given as a diagnosis by clinicians reviewing the clinical records after discharge (this secondary analysis is included in the Additional files only).

Malaria parasite status was retrospectively defined as positive if any of three slides taken over the first 3 days of admission were positive for *P. falciparum*, and the highest parasite density of these was used in analysis. Malaria mortality was defined as inpatient death in association with a positive malaria slide. We refer to mortality when describing absolute numbers of children dying with malaria, and case fatality when describing the proportion of deaths among those admitted.

### Analysis

The analysis in this study runs from May 1989 to December 2016 and was restricted to children aged 14 days to 14 years of age. Stata software was used (Version 15.0, College Station, TX: StataCorp; 2017). The binomial method was used to calculate 95% confidence intervals for median ages, and Kruskal-Wallis to compare median ages by year restricted to subgroups of children with severe malaria. Logistic regression was used to examine risk of death, including all children admitted with a positive malaria slide. Three time-periods were defined: 1989–2003, 2004–2008, and 2009–2016, covering before, during, and after the reduction in malaria cases, respectively [11]. In community surveys, declining transmission was documented beginning in the mid-1990s [10], and hence preceded the declines observed in clinical cases. We justify these three time periods as corresponding to the transition in clinical presentation to KCH, which would be expected to have a non-linear relationship with transmission intensity [9, 24]. Models were developed as follows: univariable analyses; multivariable analysis of all covariates; backwards exclusion of non-significant associations (i.e. *p* > 0.05) excepting the three major grouping of severe malaria, age, and time period which were maintained regardless of significance; then adding interactions between covariates and time (excluding those for which *p* > 0.05). We also conducted a sub-analysis which included only children resident in the KHDSS, which afforded calculation of denominators to derive incidence rates.

## Results

Between 6 May 1989 and 31 December 2016, there were 116,056 pediatric and neonatal admissions to Kilifi County Hospital (KCH), of whom 99,126 (85.4%) children were aged 14 days to 14 years. Within this age group, 17,691 (17.8%) presented with one or more criteria for severe malaria, of whom 12,805 (72.3%) had a parasite count of > 2500/μl. Annual pediatric admissions to KCH varied, from 3886 in the mid-1990s to a high of 4700 in the year 2000 before falling to 2986 in 2014 (Additional file 6: Figure S1).

### Trends in severe malaria cases

**Fig. 1:**
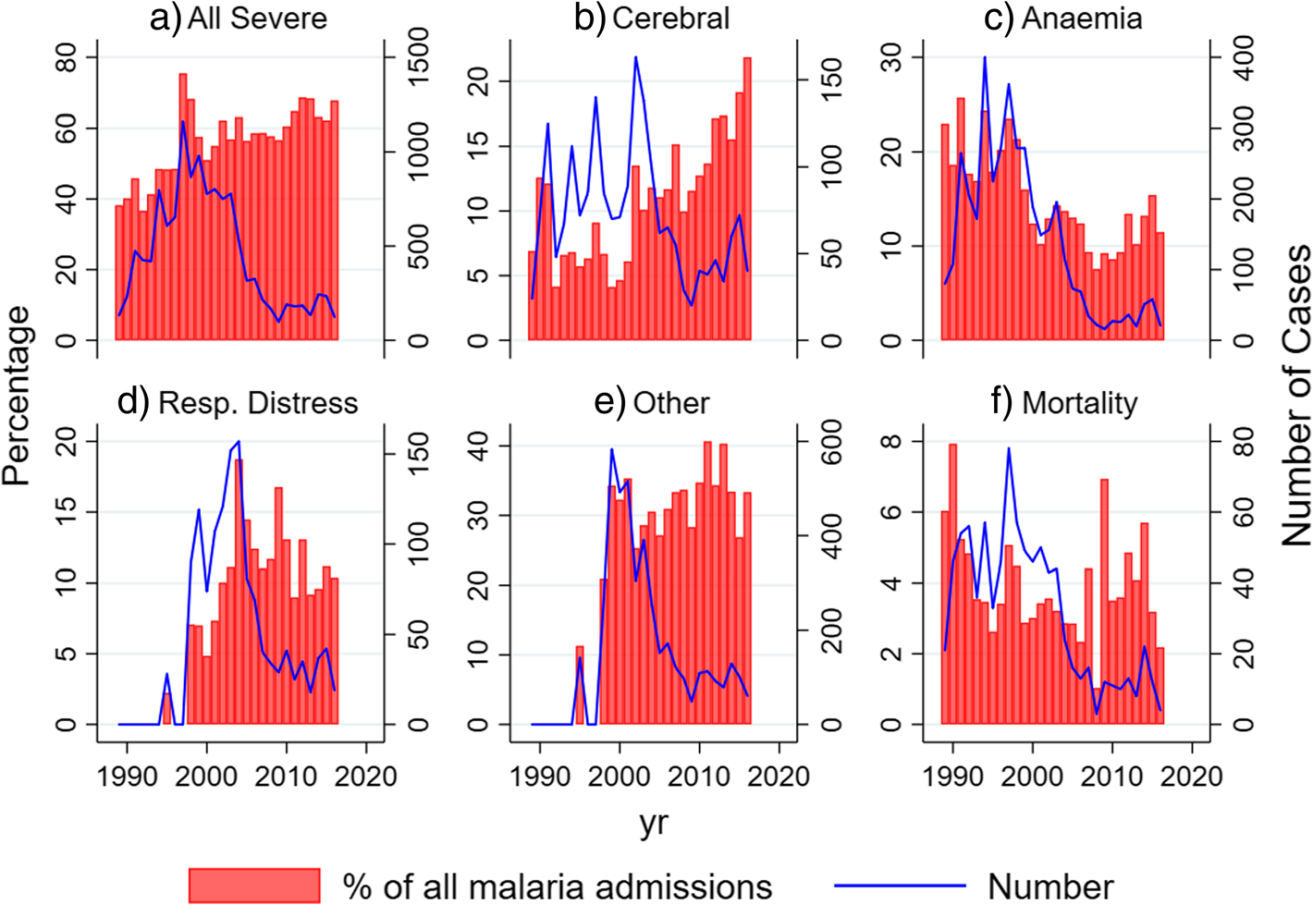
Trends in mortality and clinical features of severe malaria over time. The trends over time are shown for clinical features of severe malaria among all children admitted with a parasite threshold of > 2500 μl. The percentage of children with a parasite threshold > 2500/μl where the relevant observation was positive is shown in red bars (left *y*-axis), and the absolute number of cases where the observation was positive is shown by the blue line (right *y*-axis). **a** All severe. **b** Cerebral. **c** Anaemia. **d** Resp. distress. **e** Other. **f** Mortality

The number of severe malaria cases peaked in 1999 at 979 cases for the year. However, this apparent peak coincided with more complete data collection spanning the full range of signs and symptoms of severe malaria in 1999 (panels d and e of Fig. 1 and Additional file 6: Figure S1, Additional file 2: Table S2). Data collection was consistent from 2000 onwards to 2016: nevertheless, we observed a consistent declining trend after 2000 to a nadir at 98 cases in 2009, and a subsequent slight increase after 2009 to reach a peak of 234 cases in 2015 (Additional file 7: Figure S2).

The period 1989 to 2003 included averages of 657 cases of severe malaria per year and 48 malaria-associated deaths per year. From 2004 to 2008, there were averages of 310 cases and 14 deaths per year, and from 2009 to 2016, there were 174 cases and 12 deaths per year. The absolute numbers of non-severe malaria admissions fell more year-on-year than the absolute numbers of severe malaria admissions; hence, there was a general trend for an increasing proportion of all admissions with one or more criteria for severe malaria increased year-on-year.

### Criteria for severe malaria

There were averages of 85, 62, and 44 cases of cerebral malaria per year with 14 (16%), 10 (16%), and 8 (18%) deaths in the periods 1989–2003, 2004–2008, and 2009–2016, respectively. The proportion of children with cerebral malaria gradually changed from 7% before 2004 to 22% after 2009 (Fig. 1). Respiratory distress, hypoglycaemia, hyperparasitaemia, and multiple convulsions were experienced by > 10% of children, but jaundice, kidney injury, and compensated shock were uncommon (i.e. < 5%), with almost no cases of decompensated shock. There was no consistent trend over time in the proportion of children showing these additional criteria excepting the increase in 1999 which coincided with more complete data collection (Additional file 7: Figure S2).

### Incidence among residents of the KHDSS area

Similar trends in clinical features and mortality were seen when data were restricted to children resident within the KHDSS area, where KHDSS data afforded denominators with which to calculate incidence rates. The incidence of severe malaria fell from 7.9 per 1000 in 2003 to 1.6 per 1000 in 2015. Cerebral malaria fell from 1.3 per 1000 in 2003 to a low of 0.1 per 1000 in 2008, but then increased to 0.5 per 1000 in 2015 (Additional file 8: Figure S3).

### Trends in median age

**Fig. 2:**
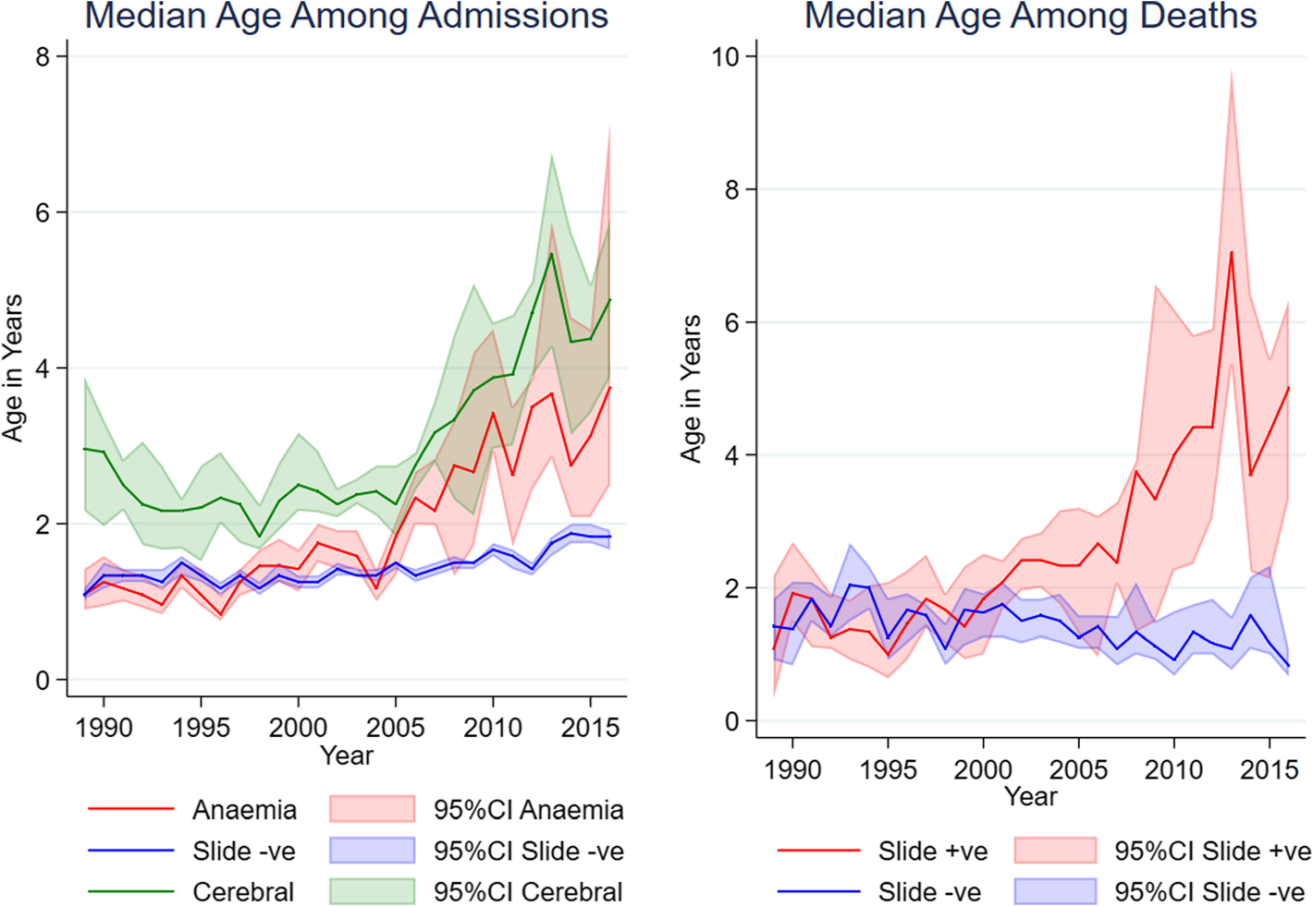
Median ages for children admitted to Kilifi County Hospital. Median ages of presentation to hospital for specific phenotypes (see color legend) are shown over time with 95% confidence intervals calculated by the binomial exact method

We studied median ages of children with different clinical features of malaria as an indication of the relative susceptibilities of older vs younger children (Fig. 2). There may be changes in the age structure of children in the general population, and other confounding secular changes in the ages of children attending hospital. We therefore compared the median ages of children with severe malaria with children hospitalized with non-malarial conditions, and compared children dying with and without malaria.

In 1989, the median ages of children in the following groups (a) hospitalized with non-malarial conditions, (b) with severe malaria anaemia, and (c) with cerebral malaria were (a) 1.1 years (95% CI 1.0–1.2), (b) 1.1 years (95% CI 0.9–1.4), and (c) 3.0 years (95% CI 2.2–3.9), respectively. By the year 2016, the median ages had increased in each of these three group to (a) 1.8 years (95% CI 1.7–1.9), (b) 3.8 years (95% CI 2.5–7.1), and (c) 4.9 years (95% CI 3.9–5.9), respectively. Hence, although there was a slight increase in median age among children hospitalized with non-malarial conditions, there was a more marked increase in median ages in those hospitalized with severe anaemia and cerebral malaria (*p* < 0.0001 for comparison of 1989 with 2016).

Among children dying with and without malaria, the median ages in 1989 were 1.1 years (95% CI 0.3–2.2) and 1.4 years (95% CI 0.9–1.8), respectively, rising by 2016 to 5 years (95% CI 3.3–6.3) and 1.2 years (95% CI 1–1.3), respectively. Similar results were seen using the alternate case definition (Additional file 9: Figure S4).

### Case fatality

Univariable analysis demonstrated that all the criteria tested for severe malaria were associated with increased risk of mortality except for prostration (Table 1). On multivariable analysis including all factors, the independent predictors of case fatality were cerebral malaria, respiratory distress, severe anaemia, acidosis, kidney injury, and hypoglycaemia (“Multivariable (all variables)” in Table 1). The next step in developing the model was to retain the criteria for clinical malaria that were statistically significant predictors, while also retaining time period, age, and cerebral/respiratory distress/severe anaemia in the model (“Multivariable (restricted)” in Table 1).

**Table 1:** Univariable and multivariable logistic regression models for risk of mortality in severe malaria (case definition includes the > 2500 parasites per μl threshold)

Predictors	Univariable		Multivariable (all variables)		Multivariable (restricted)		Interaction model	
	Odds ratio	*p* value	Odds ratio	*p* value	Odds ratio	*p* value	Odds ratio	*p* value
Time (1989–2003)	Reference							
Time (2004–2008)	.7 (.55 to .9)	0.005	NA	NA	.68 (.48 to .97)	0.03	.8 (.53 to 1.2)	0.28
Time (2009–2016)	1.09 (.88 to 1.37)	0.43	.75 (.46 to 1.24)	0.26	1.05 (.68 to 1.6)	0.83	.8 (.48 to 1.32)	0.38
Acidosis	7.07 (5.77 to 8.67)	< 0.0001	3.31 (2.09 to 5.25)	< 0.0001	2.75 (2.01 to 3.76)	< 0.0001	2.83 (2.06 to 3.89)	< 0.0001
Age (years)	.96 (.93 to .99)	0.02	1.08 (.98 to 1.18)	0.11	1.05 (.98 to 1.12)	0.19	1.05 (.98 to 1.13)	0.16
Cerebral	7.63 (6.59 to 8.83)	< 0.0001	3.81 (2.54 to 5.74)	< 0.0001	4.21 (3.17 to 5.57)	< 0.0001	4.38 (3.3 to 5.82)	< 0.0001
Compensated shock	4.76 (3.4 to 6.66)	< 0.0001	1.03 (.47 to 2.23)	0.94	NA	NA	NA	NA
Hyperparasitaemia	1.19 (1.02 to 1.38)	0.03	.89 (.58 to 1.37)	0.6	NA	NA	.75 (.51 to 1.09)	0.13
Hypoglycaemia	8.81 (6.98 to 11.1)	< 0.0001	1.93 (1.17 to 3.2)	0.01	3 (2.17 to 4.14)	< 0.0001	3.2 (2.3 to 4.45)	< 0.0001
Kidney injury	6.09 (4.43 to 8.37)	< 0.0001	2.45 (1.29 to 4.63)	0.006	2.76 (1.79 to 4.25)	< 0.0001	2.64 (1.71 to 4.09)	< 0.0001
Mx convulsions	2.21 (1.67 to 2.92)	< 0.0001	.92 (.56 to 1.51)	0.75	NA	NA	NA	NA
Jaundice	2.43 (1.49 to 3.98)	0.0004	.87 (.31 to 2.49)	0.8	NA	NA	NA	NA
Prostration	1.05 (.71 to 1.56)	0.81	NA	NA	NA	NA	NA	NA
Resp. distress	9.41 (7.69 to 11.5)	< 0.0001	2.23 (1.45 to 3.42)	0.0002	1.92 (1.42 to 2.6)	< 0.0001	1.95 (1.44 to 2.65)	< 0.0001
Severe anaemia	2.24 (1.93 to 2.6)	< 0.0001	1.25 (.78 to 1.99)	0.35	1.1 (.78 to 1.54)	0.59	1.08 (.77 to 1.52)	0.66
Time (2004–2008) * Hyperparasitaemia			NA	NA	NA	NA	.63 (.29 to 1.34)	0.23
Time (2009–2016) * Hyperparasitaemia			NA	NA	NA	NA	2.53 (1.07 to 5.97)	0.03

Footnote: Odds ratios (ORs) are shown with 95% confidence intervals in brackets. Interaction terms are not relevant to univariable models. “NA” is shown for cells where the model was not applicable, and “-” is shown for cells with insufficient data (occurring where multiple seizures and other covariates were not collected between 2004 and 2009). Multivariable (restricted) refers to a model where non-significant predictors were excluded from the model

Increasing age was associated with reduced mortality on univariable analysis, but this association was not seen on multivariable analysis. Time period was associated with variations in mortality in univariable and restricted multivariable analysis, but this association was not seen after adjusting in the interaction model. In the post-decline time period (i.e. 2009–2016), there was a slight, but statistically non-significant, increase in mortality on unadjusted analysis which was not evident after adjusting.

We selected interactions to consider for the final model by examining the unadjusted interactions between criteria for severe malaria and time period (Additional file 3: Table S3). Adjusted interactions were not significant between time period and acidosis (*p* = 0.8), cerebral malaria (*p* = 0.7), age (*p* = 0.6), compensated shock (*p* = 0.7), and hypoglycaemia (*p* = 0.5) and respiratory distress (*p* = 0.7). Age and time period did not show a significant interaction (*p* = 0.7), indicating that the case fatality for a given age and phenotype was consistent over time. These interactions with time period were not retained in the final model. The adjusted interaction was statistically significant for hyperparasitaemia (*p* = 0.001), which was retained for the final interaction model (Table 1, “Interaction Model”). Using the alternate case definition based on clinical judgement, the post-decline increase in mortality was significant when unadjusted, but again was not significant after adjusting (Additional file 4: Table S4, Additional file 5: Table S5).

The goodness of fit was estimated using Akaike’s Information Criteria scores, which were 812.2 for the multivariable (all variables) model, 804.7 for the multivariable (restricted) model and 803.6 for the interaction model with 13, 9, and 11 degrees of freedom, respectively. This indicates best fit with the interaction model.

## Discussion

Over 27 years of continuous longitudinal surveillance, we show substantial changes in the numbers of children admitted to hospital with severe malaria. We previously reported the epidemiological transition to the nadir of severe malaria cases in 2009 [12] and reported spatial and temporal distributions of malaria slide positivity through to 2014 [11]. This updated report includes more detailed clinical categorizations through to 2016.

Consistent with previous reports comparing differing malaria transmission settings [24] or previous longitudinal studies in Kilifi [12, 25], we noted increasing median ages among children with severe anaemia, cerebral malaria, and malaria-associated mortality at later time periods. In 1989, the burden of paediatric severe malaria was largely limited to children under 5 years of age. By 2016, half of all children with severe malaria were above the age of 5 years. During this period of increasing age, there was evidence of declining transmission through community surveys [26], and anti-malarial antibody levels were declining in the population [27]. The increase in median age, taken together with external evidence of falling malaria transmission, suggests that older children at later time periods had experienced less exposure to malaria during childhood leading to less immunity than children living in Kilifi District during the earlier time periods. There is some evidence that age modifies the rate of acquisition of immunity to malaria. Migrants in Indonesia moved from non-endemic to malaria endemic conditions acquired functional immunity rapidly, particularly at older ages [28]. On the other hand, infants were found to more rapidly acquire immunity than older children in randomized trials of malaria prophylaxis in Tanzania, albeit at a higher risk of developing severe malaria [29].

In longitudinal surveillance, the picture continues to evolve after a period of changing transition. For example, an area in transition from moderate to low will retain a cohort of older children who acquired immunity at moderate transmission intensity. Over time, a cohort of older children will emerge who acquired only limited immunity at low transmission intensity when they were younger, and as this cohort emerges, the average age of malaria will continue to rise and rates of admission with malaria may rise again after a nadir.

What impact does lowered immunity have on case fatality? Predictors of case fatality remained constant over time, except for hyperparasitaemia which emerged as a risk factor for case fatality after 2009. The increasing proportion of children with cerebral malaria and the emergence of hyperparasitaemia as a risk factor might have been anticipated to lead to a relative increase in case fatality of severe malaria. There was a statistically non-significant increase in case fatality after 2009 in primary analysis (Table 1), and a statistically significant increase using the alternate case definition on clinical criteria (Additional file 4: Table S4). In both cases, this increase was accounted for by adjusting for other factors and likely relates to the increased case fatality seen in cerebral malaria. However, overall numbers of admissions fell; hence, there was no increase in absolute numbers of deaths.

Cerebral malaria was associated with high case fatality throughout the monitoring period, as previously described [7]. One of the long-term complications of cerebral malaria is neurological sequelae. A meta-analysis incorporating studies with similar case definitions of cerebral malaria, indicating that sequelae occurred in approximately 11% [30]. However, these were largely in children of younger age. Few data exist on whether older age survivors of cerebral malaria experience a similar proportion of neurological sequelae.

The study has limitations. Data were incomplete, particularly for the presence of multiple convulsions, although this was not an independent predictor of mortality. Secular trends may confound our analysis. Improved medical management has already been mentioned, and improved access to community and/or hospital healthcare is likely to impact the frequency and/or severity of presentation with malaria. While fertility rates are falling, increasing child survival and changes in hospital usage might have impacts on the median age of children coming to the hospital [14]. There was a trend of increasing age among children without malaria, but no increasing age among fatal cases (Fig. 2), suggesting that the increases in median age among children with malaria are not due to a general trend impacting the wider population. It is possible that other trends countered the potential impact on case fatality such as improved medical care with intravenous artesunate in 2010 [6] or improved fluid management [31]. We did not undertake genotyping to distinguish autochthonous from imported cases, although a previous analysis of human and parasite movement in 2009 suggests that most cases are autochthonous [32].

Defining the true burden of severe malaria is complex including the poor specificity of case definitions that include asymptomatic parasitaemia that coincide with a child presenting with severe illness due to another underlying non-malarial etiology [23]. We mitigated this misclassification by using two different case definitions: (a) including a parasite density threshold and (b) restricting analysis to those children in whom clinicians had confirmed a discharge diagnosis of malaria. In other studies, this has been examined using quantitative plasma *Plasmodium falciparum* histidine-rich protein 2 as a proxy for the sequestered parasite biomass [33]. Our definitions of severe malarial anaemia and cerebral malaria followed the WHO definition [21] but could have been refined using additional clinical criteria [7, 34] and retinal examination [35], which we did not conduct. We did not see a consistent trend over time for parasite densities (Additional file 10: Figure S5), and the majority of parasite densities were above the threshold we applied. This threshold was previously found to have consistent specificity and sensitivity across a range of transmission settings [23], and we found consistent results when we did not apply a threshold (Additional file 8: Figure S3 and Additional file 4: Table S4).

## Conclusion

Our data indicate one possible outcome of a secular trend of reducing malaria transmission followed by stagnation, and this secular trend seems to be widespread in many parts of Africa [4]. The transition to a predominance of the cerebral malaria phenotype with high case fatalities is a concern and should stimulate research and clinical trials aimed at improving outcome for this complication. Investment in data collection in routine settings is essential to more widely describe the outcomes of changes in malaria transmission and access to care and offers a cost-effective and scalable solution to malaria monitoring.

## Additional files

Additional file 1: **Table S1.** Prevalence of asymptomatic parasitaemia among children admitted with trauma. (DOCX 12 kb)

Additional file 2: **Table S2.** Frequency of mortality and criteria for severe malaria by Year. (DOCX 17 kb)

Additional file 3: **Table S3.** Unadjusted logistic regression for risk of death by time period. Case definition includes parasite density threshold (i.e. > 2500 parasites per μl). (DOCX 14 kb)

Additional file 4: **Table S4.** Unadjusted logistic regression for risk of death by time period. Case definition includes diagnosis by clinician. (DOCX 14 kb)

Additional file 5: **Table S5.** Univariate and multivariate logistic regression models for risk of mortality in severe malaria. Case definition includes diagnosis by clinician. (DOCX 14 kb)

Additional file 6: **Figure S1.** Numbers of admissions to Kilifi County Hospital over time. The percentage of admissions drawn from the Kilifi Health Demographic Surveillance System are shown on the left Y axis. Total annual admissions are shown on the right Y axis. The KHDSS was established in 2001, hence earlier residence in the DSS is reconstructed based on the reported location of residence in the clinical record. Hence the appearance of an expansion in KHDSS residence after 2001 is likely to reflect greater ascertainment of residence. (PNG 151 kb)

Additional file 7: **Figure S2.** Trends in mortality, full clinical features of severe malaria and completeness of data collection over time. The trends over time are shown for clinical features of severe malaria (panels b-n) and mortality (panel a); giving the % of all admissions where a relevant observation for the panel subtitle was made (green bars, left y axis); the % of all admissions where the relevant observation was positive (red bar, left y axis); and the absolute number of cases where the observation was positive (blue line). (PNG 150 kb)

Additional file 8: **Figure S3.** Trends in incidence of mortality and clinical features of severe malaria over time for Kilifi Health and Demographic Surveillance System Residents. The trends over time are shown for clinical features of severe malaria with a parasite threshold of > 2500 (panels a-e) and mortality (panel f); giving the % of all admissions where the relevant observation was positive (red bars, left y axis); and the incidence of cases per 1000 population among under 14 year old children (blue line). (PNG 124 kb)

Additional file 9: **Figure S4.** Median ages for children admitted to Kilifi County Hospital. Case definition includes diagnosis by clinician. Median ages of presentation to hospital for specific phenotypes (see color legend) are shown over time with 95% confidence intervals calculated by the binomial exact method. (PNG 152 kb)

Additional file 10: **Figure S5.** Parasite densities by year. The box plots show median parasite densities, interquartile ranges with boxes and adjacent values (i.e. lowest and highest observations within 1.5 times the interquartile range) with whiskers. Outlying values are shown by circles. (PNG 112 kb)

## Data Availability

Data and analysis files are available in Harvard Dataverse at https://doi.org/10.7910/DVN/2HCFW0 under managed access given the joint ownership of data with the County Hospital. Applications for access can be made through the Data Governance Committee with details available on www.kemri-wellcome.org, or on email to cgmrc@kemri-wellcome.org.

## Abbreviations

HDU: High dependency unit
KCH: Kilifi County Hospital
KEMRI SERU: Kenya Medical Research Institute Science and Ethics Review Unit
KEMRI SSC/ERC: Kenya Medical Research Institute Scientific Steering Committee/ Ethical Review Committee
KHDSS: Kilifi Health and Demographic Surveillance System
pRIFLE: Paediatric risk, injury, failure, loss, and end-stage renal disease
sSA: Sub-Saharan Africa
WHO: World Health Organization
