# Supplementary material for: Observational study: 27 years of severe malaria surveillance in Kilifi, Kenya
Source: BMC Med. 2019 Jul 8;17:124. doi: 10.1186/s12916-019-1359-9 (PMC6613255; doi:10.1186/s12916-019-1359-9)
Supplement: Supplementary file 1 — Table S1. Prevalence of asymptomatic parasitaemia among children admitted with trauma. (DOCX 12 kb) [file 12916_2019_1359_MOESM1_ESM.docx]

Table S1: Prevalence of Asymptomatic Parasitaemia Among Children Admitted with Trauma

| Year | Prevalence (% and n/N) |
| --- | --- |
| 2000 | 50% (8/16) |
| 2001 | 35% (20/57) |
| 2002 | 31.4% (17/54) |
| 2003 | 40.6% (35/86) |
| 2004 | 22.9% (22/96) |
| 2005 | 20.8% (20/96) |
| 2006 | 11.9% (9/75) |
| 2007 | 7.6% (7/91) |
| 2008 | 3.2% (3/91) |
| 2009 | .8% (1/113) |
| 2010 | 1.1% (1/85) |
| 2011 | 2.2% (2/87) |
| 2012 | 5.3% (4/75) |
| 2013 | 2.1% (2/93) |
| 2014 | 2.1% (2/95) |
| 2015 | 10.3% (11/106) |
| 2016 | 9.8% (11/112) |

Children admitted for acute traumatic injury in the absence of fever or prior febrile symptoms are used to reflect the prevalence of asymptomatic parasitaemia in the community from which admissions are drawn. The % prevalence and n/N for numerator/denominator are given by year.
