# Supplementary material for: Observational study: 27 years of severe malaria surveillance in Kilifi, Kenya
Source: BMC Med. 2019 Jul 8;17:124. doi: 10.1186/s12916-019-1359-9 (PMC6613255; doi:10.1186/s12916-019-1359-9)
Supplement: Supplementary file 2 — Table S2. Frequency of mortality and criteria for severe malaria by Year. (DOCX 17 kb) [file 12916_2019_1359_MOESM2_ESM.docx]

Table S2: Frequency of Mortality and Criteria for Severe Malaria by Year

| Year | All Severe | Mortality | Anaemia | Cerebral | Resp | Acidosis | Comp Shock | Hyperpara | Hypoglyc | Renal | Mx Conv | Jaundice | Prostrate |
| --- | --- | --- | --- | --- | --- | --- | --- | --- | --- | --- | --- | --- | --- |
| 1989 | 133/348 (38.2%) | 21/348 (6%) | 80/343 (23.2%) | 24/348 (6.90%) | - | - | - | 46/348 (13.1%) | - | - | - | - | - |
| 1990 | 233/580 (40.2%) | 46/580 (7.90%) | 108/577 (18.7%) | 73/580 (12.6%) | - | - | - | 97/580 (16.7%) | - | - | - | - | - |
| 1991 | 473/1031 (45.9%) | 54/1031 (5.19%) | 265/996 (26.6%) | 125/1013 (12.3%) | - | - | - | 149/1031 (14.5%) | - | - | - | - | - |
| 1992 | 425/1159 (36.7%) | 56/1159 (4.80%) | 205/1126 (18.2%) | 48/1048 (4.59%) | - | - | - | 232/1159 (20%) | - | - | - | - | - |
| 1993 | 420/1016 (41.2%) | 36/1016 (3.5%) | 172/997 (17.2%) | 67/999 (6.69%) | - | 19/52 (36.5%) | - | 218/1016 (21.5%) | - | - | - | - | - |
| 1994 | 797/1643 (48.5%) | 57/1642 (3.5%) | 400/1621 (24.7%) | 112/1599 (7%) | - | 51/108 (47.2%) | - | 404/1643 (24.6%) | - | - | - | - | - |
| 1995 | 608/1257 (48.4%) | 33/1250 (2.59%) | 225/1249 (18%) | 72/1245 (5.80%) | 28/507 (5.5%) | 44/108 (40.7%) | - | 329/1257 (26.2%) | - | - | - | - | 17/506 (3.40%) |
| 1996 | 653/1345 (48.5%) | 46/1341 (3.40%) | 272/1340 (20.2%) | 85/1327 (6.40%) | - | 51/104  (49%) | - | 349/1345 (25.8%) | - | - | - | - | - |
| 1997 | 1161/1539 (75.4%) | 78/1539 (5.09%) | 362/1533 (23.6%) | 140/1519 (9.19%) | - | 96/206 (46.5%) | - | 439/1539 (28.5%) | - | - | - | - | - |
| 1998 | 868/1273 (68.1%) | 57/1273 (4.5%) | 272/1267 (21.5%) | 85/1267 (6.69%) | 90/813 (11.1%) | 101/228 (44.2%) | - | 234/1273 (18.3%) | 36/335 (10.6%) | 31/371 (8.39%) | 155/1273 (12.1%) | 18/818 (2.20%) | 70/813 (8.60%) |
| 1999 | 979/1700 (57.5%) | 49/1700 (2.90%) | 272/1697 (16%) | 70/1700 (4.09%) | 119/1699 (7%) | 167/384 (43.5%) | 0/268  (0%) | 356/1700 (20.8%) | 95/822 (11.6%) | 40/472 (8.5%) | 213/1700 (12.5%) | 20/1699 (1.20%) | 140/1699 (8.19%) |
| 2000 | 778/1525 (51%) | 46/1525 (3%) | 189/1522 (12.3%) | 71/1525 (4.69%) | 74/1525 (4.90%) | 165/752 (21.8%) | 0/1525  (0%) | 332/1525 (21.7%) | 46/509 (9%) | 33/873 (3.79%) | 145/1525 (9.5%) | 24/1525 (1.60%) | 101/1525 (6.59%) |
| 2001 | 802/1458 (55%) | 50/1457 (3.40%) | 149/1456 (10.1%) | 89/1458 (6.09%) | 107/1456 (7.30%) | 272/1091 (24.8%) | 0/1458  (0%) | 305/1458 (20.8%) | 59/563 (10.5%) | 37/684 (5.40%) | 96/1458 (6.59%) | 18/1456 (1.20%) | 107/1456 (7.30%) |
| 2002 | 750/1206 (62.2%) | 43/1206 (3.59%) | 156/1200 (13%) | 163/1204 (13.5%) | 121/870 (13.8%) | 261/1129 (23.1%) | 59/1204  (4.90%) | 264/1206 (21.8%) | 53/973 (5.40%) | 30/956 (3.09%) | 153/1204 (12.6%) | 14/870 (1.60%) | 22/870 (2.5%) |
| 2003 | 778/1368 (56.9%) | 44/1368 (3.20%) | 196/1360 (14.3%) | 138/1367 (10.1%) | 152/1367 (11.1%) | 243/1211 (20.1%) | 69/1368  (5%) | 318/1368 (23.2%) | 82/1305 (6.30%) | 23/968 (2.40%) | - | 27/1367 (2%) | 46/1367 (3.40%) |
| 2004 | 529/838 (63.0%) | 24/838 (2.90%) | 115/829 (13.8%) | 99/837 (11.8%) | 157/837 (18.7%) | 149/817 (18.2%) | 70/838  (8.39%) | 200/838 (23.8%) | 53/811 (6.5%) | 17/435 (3.90%) | - | 13/837 (1.60%) | 27/837 (3.20%) |
| 2005 | 316/560 (56.4%) | 16/560 (2.90%) | 73/554 (13.1%) | 62/560 (11.1%) | 81/560 (14.5%) | 69/359 (19.2%) | 47/560  (8.39%) | 129/560 (23%) | 24/548 (4.40%) | 9/248 (3.59%) | - | 6/560 (1.10%) | 23/560 (4.09%) |
| 2006 | 326/556 (58.5%) | 13/556 (2.29%) | 69/546 (12.6%) | 65/556 (11.6%) | 69/556 (12.3%) | 69/219 (31.5%) | 19/556  (3.40%) | 178/556 (32%) | 33/550 (6%) | 11/274 (4%) | - | 10/556 (1.79%) | 24/556 (4.30%) |
| 2007 | 213/363 (58.7%) | 16/363 (4.40%) | 34/360 (9.39%) | 55/363 (15.1%) | 40/363  (11%) | 21/100 (21%) | 19/363  (5.19%) | 128/363 (35.2%) | 16/289 (5.5%) | 4/171 (2.29%) | - | 7/363 (1.89%) | 13/363 (3.59%) |
| 2008 | 168/291 (57.7%) | 3/291 (1%) | 22/289 (7.59%) | 29/290  (10%) | 34/290 (11.6%) | 20/75 (26.7%) | 8/291 (2.70%) | 84/291 (28.8%) | 24/163 (14.6%) | 1/85 (1.20%) | - | 7/290 (2.40%) | 11/290 (3.79%) |
| 2009 | 98/173 (56.5%) | 12/173 (6.90%) | 16/172 (9.30%) | 20/173 (11.6%) | 29/173 (16.7%) | 24/71 (33.7%) | 7/173 (4%) | 48/173 (27.7%) | 6/166 (3.59%) | 1/3 (33.2%) | - | 2/173 (1.20%) | 9/173 (5.19%) |
| 2010 | 190/314 (60.5%) | 11/314 (3.5%) | 27/313 (8.60%) | 40/312 (12.8%) | 41/312 (13.1%) | 31/139 (22.2%) | 11/314 (3.5%) | 91/314 (29%) | 6/306 (2%) | 3/81 (3.70%) | - | 14/312 (4.5%) | 29/312 (9.30%) |
| 2011 | 180/278 (64.6%) | 10/278 (3.59%) | 26/277 (9.39%) | 38/278 (13.6%) | 25/278  (9%) | 27/125 (21.6%) | 4/278 (1.39%) | 91/278 (32.7%) | 10/264 (3.79%) | 1/15 (6.69%) | 42/260 (16.2%) | 11/278 (4%) | 19/278 (6.80%) |
| 2012 | 184/268 (68.6%) | 13/268 (4.90%) | 36/266 (13.5%) | 46/268 (17.2%) | 35/268 (13.1%) | 38/134 (28.3%) | 3/268 (1.10%) | 74/268 (27.6%) | 17/256 (6.59%) | 1/49 (2%) | 36/268 (13.3%) | 4/268 (1.5%) | 23/268 (8.60%) |
| 2013 | 134/196 (68.4%) | 8/196 (4.09%) | 20/194 (10.3%) | 34/196 (17.2%) | 18/196 (9.19%) | 11/107 (10.3%) | 1/196 (.5%) | 44/196 (22.3%) | 3/167 (1.79%) | 1/98 (1%) | 28/196 (14.3%) | 6/196 (3.09%) | 31/196 (15.8%) |
| 2014 | 244/386 (63.2%) | 22/386 (5.69%) | 51/380 (13.3%) | 60/385 (15.6%) | 37/385 (9.60%) | 43/166 (25.8%) | 9/386 (2.29%) | 83/386 (21.5%) | 14/366 (3.79%) | 1/320 (.300%) | 49/385 (12.6%) | 14/385 (3.59%) | 50/385 (13%) |
| 2015 | 234/376 (62.2%) | 12/376 (3.20%) | 58/373 (15.5%) | 72/376 (19.1%) | 42/376 (11.1%) | 28/139 (20.1%) | 12/376 (3.20%) | 74/376 (19.7%) | 6/362 (1.70%) | 2/351 (.600%) | 51/376 (13.6%) | 20/376 (5.30%) | 30/376 (8%) |
| 2016 | 124/183 (67.8%) | 4/183 (2.20%) | 21/182 (11.5%) | 40/183 (21.8%) | 19/183 (10.3%) | 11/77 (14.3%) | 0/183 (0%) | 41/183 (22.3%) | 8/165 (4.80%) | 2/177 (1.10%) | 21/183 (11.5%) | 7/183 (3.79%) | 16/183 (8.69%) |

Footnote: Cells show numerator over denominator (i.e. number of children with the clinical feature indicated in the column over all children where data were collected) and percentage in brackets. “-“ indicates that no data were collected. Abbreviations: All Severe - All cases of severe malaria; Anaemia – Severe Malaria Anaemia; Cerebral – Cerebral Malaria; Resp – Respiratory Distress; Comp Shock – Compensated Shock; Hyperpara – Hyperparasitaemia; Hypoglc – Hypoglycemia; Renal – Renal Injury; Mx Conv – Multiple Convulsions.
