# Supplementary material for: Observational study: 27 years of severe malaria surveillance in Kilifi, Kenya
Source: BMC Med. 2019 Jul 8;17:124. doi: 10.1186/s12916-019-1359-9 (PMC6613255; doi:10.1186/s12916-019-1359-9)
Supplement: Supplementary file 3 — Table S3. Unadjusted logistic regression for risk of death by time period. Case definition includes parasite density threshold (i.e. > 2500 parasites per μl). (DOCX 14 kb) [file 12916_2019_1359_MOESM3_ESM.docx]

Table S3: Unadjusted Logistic Regression for Risk of Death by Time Period. Case Definition Includes Parasite Density threshold (i.e. >2,500 Parasites Per µl).

| **Predictor** | **1989** | | **2004** | | **2009** | | **Interaction with time P** |
| --- | --- | --- | --- | --- | --- | --- | --- |
| **Case definition >2,500 parasites per µl** | **Odds Ratio** | **P** | **Odds Ratio** | **P** | **Odds Ratio** | **P** |  |
| Acidosis | 10.6 (8.24 to 13.8) | <0.0001 | 6.64 (4.2 to 10.5) | <0.0001 | 3.79 (2.57 to 5.61) | <0.0001 | 0.0001 |
| Cerebral Malaria | 9.62 (8.15 to 11.3) | <0.0001 | 15.6 (10.1 to 24) | <0.0001 | 8.24 (5.64 to 12.0) | <0.0001 | 0.07 |
| Anaemia + Respiratory | 13.4 (9.68 to 18.6) | <0.0001 | 6.87 (4.02 to 11.7) | <0.0001 | 6.41 (3.75 to 10.9) | <0.0001 | 0.02 |
| Anaemia + Cerebral | 8.51 (6.44 to 11.2) | <0.0001 | 8.49 (4.47 to 16.1) | <0.0001 | 8.26 (4.78 to 14.2) | <0.0001 | 0.99 |
| Cerebral + Respiratory | 18.6 (13.4 to 25.7) | <0.0001 | 12.2 (7.94 to 19.0) | <0.0001 | 11.0 (7.41 to 16.5) | <0.0001 | 0.1 |
| Cerebral + Respiratory + Anaemia | 16.9 (9.36 to 30.5) | <0.0001 | 9.11 (4.4 to 18.8) | <0.0001 | 12.9 (6.68 to 25.2) | <0.0001 | 0.43 |
| Compensated Shock | 6.92 (4.38 to 10.9) | <0.0001 | 4 (2.42 to 6.61) | <0.0001 | 2.38 (1.01 to 5.61) | 0.05 | 0.06 |
| Hyperparasitaemia | 1.46 (1.22 to 1.75) | <0.0001 | .96 (.58 to 1.58) | 0.86 | 1.94 (1.29 to 2.9) | 0.001 | 0.09 |
| Hypoglycaemia | 9.7 (7.3 to 12.9) | <0.0001 | 10.5 (6.66 to 16.8) | <0.0001 | 4.39 (2.41 to 7.98) | <0.0001 | 0.03 |
| Kidney Injury | 5.99 (4.28 to 8.38) | <0.0001 | 7.59 (4.01 to 14.3) | <0.0001 | 7.19 (2.59 to 19.9) | 0.0001 | 0.79 |
| Multiple Convulsions | 3.13 (2.36 to 4.17) | <0.0001 | NA | NA | 1.45 (.87 to 2.43) | 0.16 | NA |
| Jaundice | 2.5 (1.34 to 4.66) | 0.004 | 2.92 (1.14 to 7.44) | 0.03 | 1.98 (1.01 to 3.86) | 0.05 | 0.78 |
| Prostrate | 1.57 (1.01 to 2.42) | 0.04 | 1 (1 to 1) | NA | .58 (.28 to 1.2) | 0.14 | NA |
| Respiratory Distress | 13.1 (10.2 to 16.9) | <0.0001 | 9.59 (6.33 to 14.5) | <0.0001 | 7.41 (5.12 to 10.7) | <0.0001 | 0.04 |
| Severe Anaemia | 2.41 (2.05 to 2.83) | <0.0001 | 2.6 (1.65 to 4.09) | <0.0001 | 2.65 (1.77 to 3.98) | <0.0001 | 0.88 |

Footnote: Odds Ratios for death are shown in cells with 95% confidence intervals in brackets. P values are shown for unadjusted analyses in the 3^rd^, 5^th^ and 7^th^ columns. The final column shows the p value for a log likelihood test of the interaction between time and the predictor in column 1 in determining death as the outcome.
