# Supplementary material for: Observational study: 27 years of severe malaria surveillance in Kilifi, Kenya
Source: BMC Med. 2019 Jul 8;17:124. doi: 10.1186/s12916-019-1359-9 (PMC6613255; doi:10.1186/s12916-019-1359-9)
Supplement: Supplementary file 4 — Table S4. Unadjusted logistic regression for risk of death by time period. Case definition includes diagnosis by clinician. (DOCX 14 kb) [file 12916_2019_1359_MOESM4_ESM.docx]

Table S4: Unadjusted Logistic Regression for Risk of Death by Time Period. Case Definition Includes Diagnosis by Clinician.

| **Predictor** | **1989** | | **2004** | | **2009** | | **Interaction with time P** |
| --- | --- | --- | --- | --- | --- | --- | --- |
| **Case definition clinician diagnosis** | **Odds Ratio** | **P** | **Odds Ratio** | **P** | **Odds Ratio** | **P** |  |
| Acidosis | 8.1 (6.28 to 10.4) | <0.0001 | 6.26 (3.68 to 10.6) | <0.0001 | 4.98 (3.1 to 8) | <0.0001 | 0.18 |
| Cerebral Malaria | 6.67 (5.63 to 7.92) | <0.0001 | 21.3 (12.6 to 36.0) | <0.0001 | 11.9 (7.61 to 18.7) | <0.0001 | <0.0001 |
| Anaemia + Respiratory | 9.41 (6.48 to 13.6) | <0.0001 | 5.26 (2.67 to 10.3) | <0.0001 | 8.96 (4.86 to 16.5) | <0.0001 | 0.31 |
| Anaemia + Cerebral | 6.66 (4.93 to 9) | <0.0001 | 9.03 (4.19 to 19.4) | <0.0001 | 11.1 (5.83 to 21.1) | <0.0001 | 0.33 |
| Cerebral + Respiratory | 14.5 (10.1 to 20.6) | <0.0001 | 12.5 (7.57 to 20.9) | <0.0001 | 14.9 (9.23 to 24.1) | <0.0001 | 0.87 |
| Cerebral + Respiratory + Anaemia | 15.2 (7.62 to 30.6) | <0.0001 | 10.2 (4.3 to 24.5) | <0.0001 | 21.6 (9.42 to 49.9) | <0.0001 | 0.47 |
| Compensated Shock | 7.54 (4.72 to 12.0) | <0.0001 | 4.25 (2.35 to 7.68) | <0.0001 | 2.79 (1.08 to 7.23) | 0.03 | 0.1 |
| Hyperparasitaemia | 1.12 (.94 to 1.33) | 0.2 | 1.01 (.6 to 1.71) | 0.97 | 2.08 (1.35 to 3.19) | 0.0008 | 0.03 |
| Hypoglycaemia | 9.42 (7.03 to 12.6) | <0.0001 | 10.3 (6.1 to 17.4) | <0.0001 | 5.41 (2.79 to 10.5) | <0.0001 | 0.25 |
| Kidney Injury | 5.4 (3.74 to 7.78) | <0.0001 | 8.3 (3.94 to 17.5) | <0.0001 | 8.05 (2.11 to 30.8) | 0.002 | 0.55 |
| Multiple Convulsions | 2.36 (1.72 to 3.23) | <0.0001 | NA | NA | 1.72 (.94 to 3.16) | 0.08 | NA |
| Jaundice | 2.18 (1.05 to 4.53) | 0.04 | 3.76 (1.31 to 10.8) | 0.01 | 1.97 (.83 to 4.66) | 0.12 | 0.64 |
| Prostrate | 1.33 (.84 to 2.12) | 0.23 | 1 (1 to 1) | NA | .78 (.36 to 1.72) | 0.54 | NA |
| Respiratory Distress | 10.1 (7.83 to 13.2) | <0.0001 | 8.53 (5.28 to 13.8) | <0.0001 | 9.61 (6.21 to 14.8) | <0.0001 | 0.82 |
| Severe Anaemia | 2.17 (1.85 to 2.56) | <0.0001 | 2.13 (1.2 to 3.75) | 0.009 | 2.98 (1.84 to 4.81) | <0.0001 | 0.48 |

Footnote: Odds Ratios for death are shown in cells with 95% confidence intervals in brackets. P values are shown for unadjusted analyses in the 3^rd^, 5^th^ and 7^th^ columns. The final column shows the p value for a log likelihood test of the interaction between time and the predictor in column 1 in determining death as the outcome.
