# Supplementary material for: Observational study: 27 years of severe malaria surveillance in Kilifi, Kenya
Source: BMC Med. 2019 Jul 8;17:124. doi: 10.1186/s12916-019-1359-9 (PMC6613255; doi:10.1186/s12916-019-1359-9)
Supplement: Supplementary file 5 — Table S5. Univariate and multivariate logistic regression models for risk of mortality in severe malaria. Case definition includes diagnosis by clinician. (DOCX 14 kb) [file 12916_2019_1359_MOESM5_ESM.docx]

Table S5: Univariate and Multivariate Logistic Regression Models for Risk of Mortality in Severe Malaria. Case Definition Includes Diagnosis by Clinician.

| Predictors | Univariate | | | Multivariate (all variables) | | | Multivariate (restricted) | | | Interaction model | | |
| --- | --- | --- | --- | --- | --- | --- | --- | --- | --- | --- | --- | --- |
|  | Odds Ratio | P value | | Odds Ratio | | P value | Odds Ratio | | P value | Odds Ratio | | P value |
| Time (1989-2003) | Reference |  | |  | |  |  | |  |  | |  |
| Time (2004-2008) | .96 (.77 to 1.19) | 0.69 | | NA | | NA | .95 (.69 to 1.3) | | 0.73 | 1.16 (.81 to 1.65) | | 0.43 |
| Time (2009-2016) | 1.31 (1.08 to 1.59) | 0.006 | | .84 (.54 to 1.3) | | 0.43 | 1.02 (.71 to 1.48) | | 0.91 | .89 (.59 to 1.34) | | 0.58 |
| Acidosis | 7.52 (6.23 to 9.08) | <0.0001 | | 3.65 (2.38 to 5.59) | | <0.0001 | 3.35 (2.5 to 4.49) | | <0.0001 | 3.38 (2.51 to 4.54) | | <0.0001 |
| Age (years) | 1 (.97 to 1.03) | 0.77 | | 1.07 (.99 to 1.16) | | 0.11 | 1.07 (1 to 1.14) | | 0.04 | 1.07 (1.01 to 1.14) | | 0.03 |
| Cerebral | 9.58 (8.33 to 11.0) | <0.0001 | | 4.47 (3.07 to 6.52) | | <0.0001 | 4.62 (3.56 to 6) | | <0.0001 | 4.77 (3.67 to 6.2) | | <0.0001 |
| Compensated Shock | 4.41 (3.24 to 6.01) | <0.0001 | | .71 (.34 to 1.49) | | 0.36 | NA | | NA | NA | | NA |
| Hyperparasitaemia | 1.44 (1.24 to 1.69) | <0.0001 | | 1.25 (.82 to 1.92) | | 0.3 | NA | | NA | 1 (.69 to 1.46) | | 1 |
| Hypoglycaemia | 8.45 (6.79 to 10.5) | <0.0001 | | 2.01 (1.24 to 3.25) | | 0.005 | 2.87 (2.11 to 3.89) | | <0.0001 | 2.93 (2.15 to 4.01) | | <0.0001 |
| Kidney Injury | 6.41 (4.83 to 8.51) | <0.0001 | | 2.51 (1.44 to 4.36) | | 0.001 | 2.65 (1.81 to 3.89) | | <0.0001 | 2.6 (1.77 to 3.82) | | <0.0001 |
| Mx Convulsions | 2.6 (2.03 to 3.34) | <0.0001 | | 1.09 (.71 to 1.69) | | 0.69 | NA | | NA | NA | | NA |
| Jaundice | 2.52 (1.67 to 3.79) | <0.0001 | | 1.57 (.7 to 3.53) | | 0.28 | NA | | NA | NA | | NA |
| Prostration | 1.01 (.7 to 1.46) | 0.96 | | NA | | NA | NA | | NA | NA | | NA |
| Resp. Distress | 10.7 (8.93 to 12.9) | <0.0001 | | 2.13 (1.44 to 3.15) | | 0.0001 | 1.93 (1.46 to 2.54) | | <0.0001 | 1.95 (1.48 to 2.57) | | <0.0001 |
| Severe Anaemia | 2.41 (2.1 to 2.78) | | <0.0001 | | 1.72 (1.14 to 2.59) | 0.009 | | 1.44 (1.07 to 1.94) | 0.02 | | 1.43 (1.06 to 1.92) | 0.02 |
| Time (2004-2008) * Hyperpara | | | | | NA | NA | | NA | NA | | 2.25 (.97 to 5.22) | 0.06 |
| Time (2009-2016) * Hyperpara | | | | | NA | NA | | NA | NA | | NA | NA |

Unadjusted interactions seen to be significant in supplementary table 3 were not significant on adjusting; i.e. between time and cerebral malaria (p=0.16), age (p=0.9), compensated shock (p=0.8) and hypoglycaemia (p=0.4) and respiratory distress (p=0.2), and were therefore not included in the final model. The adjusted interaction was statistically significant for hyperparasitaemia (p<0.001), which is retained. The final interaction model included age as a linear variable as categorization was not statistically an improved fit (p=0.2).
