# Supplementary figures and images for: Observational study: 27 years of severe malaria surveillance in Kilifi, Kenya
Source: BMC Med. 2019 Jul 8;17:124. doi: 10.1186/s12916-019-1359-9 (PMC6613255; doi:10.1186/s12916-019-1359-9)

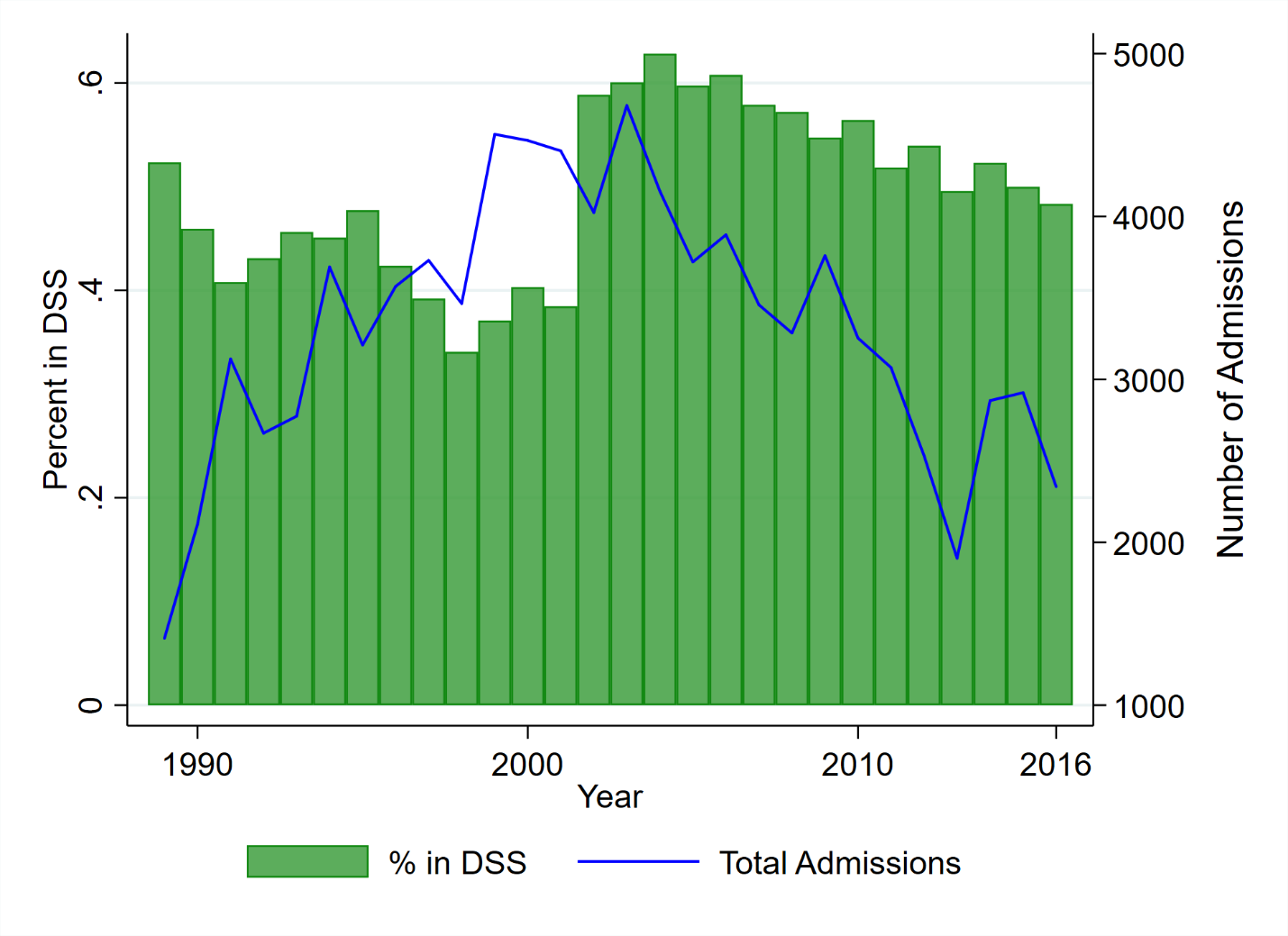

Supplement: Supplementary file 6 — Figure S1. Numbers of admissions to Kilifi County Hospital over time. The percentage of admissions drawn from the Kilifi Health Demographic Surveillance System are shown on the left Y axis. Total annual admissions are shown on the right Y axis. The KHDSS was established in 2001, hence earlier residence in the DSS is reconstructed based on the reported location of residence in the clinical record. Hence the appearance of an expansion in KHDSS residence after 2001 is likely to reflect greater ascertainment of residence. (PNG 151 kb) [file 12916_2019_1359_MOESM6_ESM.png]

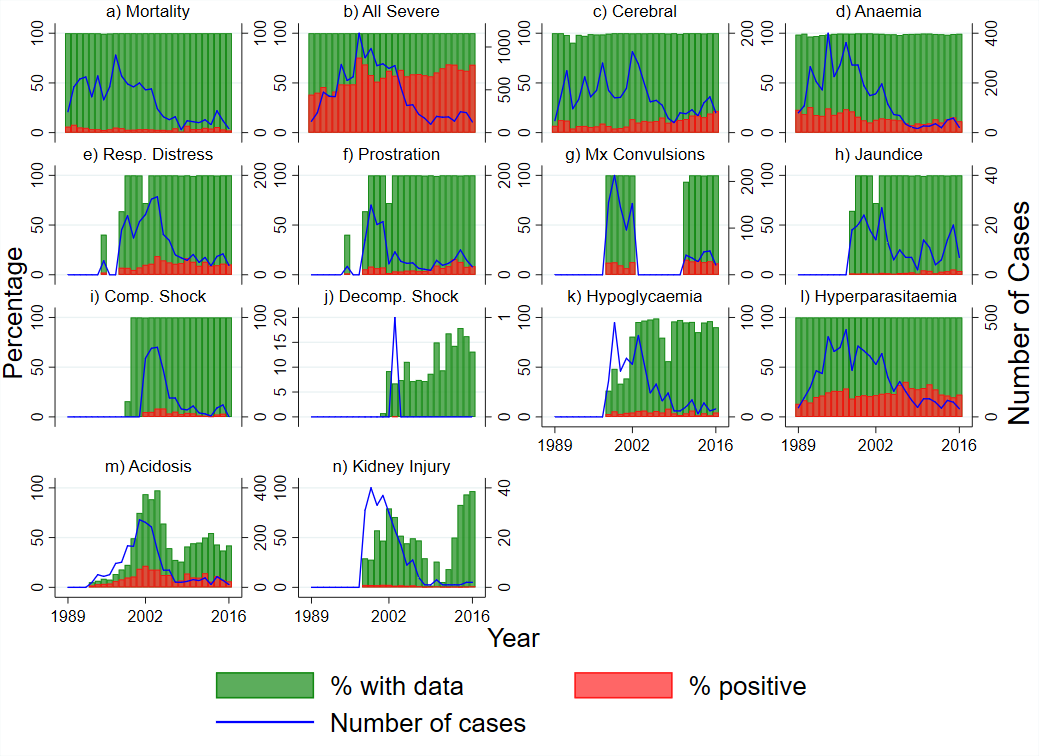

Supplement: Supplementary file 7 — Figure S2. Trends in mortality, full clinical features of severe malaria and completeness of data collection over time. The trends over time are shown for clinical features of severe malaria (panels b-n) and mortality (panel a); giving the % of all admissions where a relevant observation for the panel subtitle was made (green bars, left y axis); the % of all admissions where the relevant observation was positive (red bar, left y axis); and the absolute number of cases where the observation was positive (blue line). (PNG 150 kb) [file 12916_2019_1359_MOESM7_ESM.png]

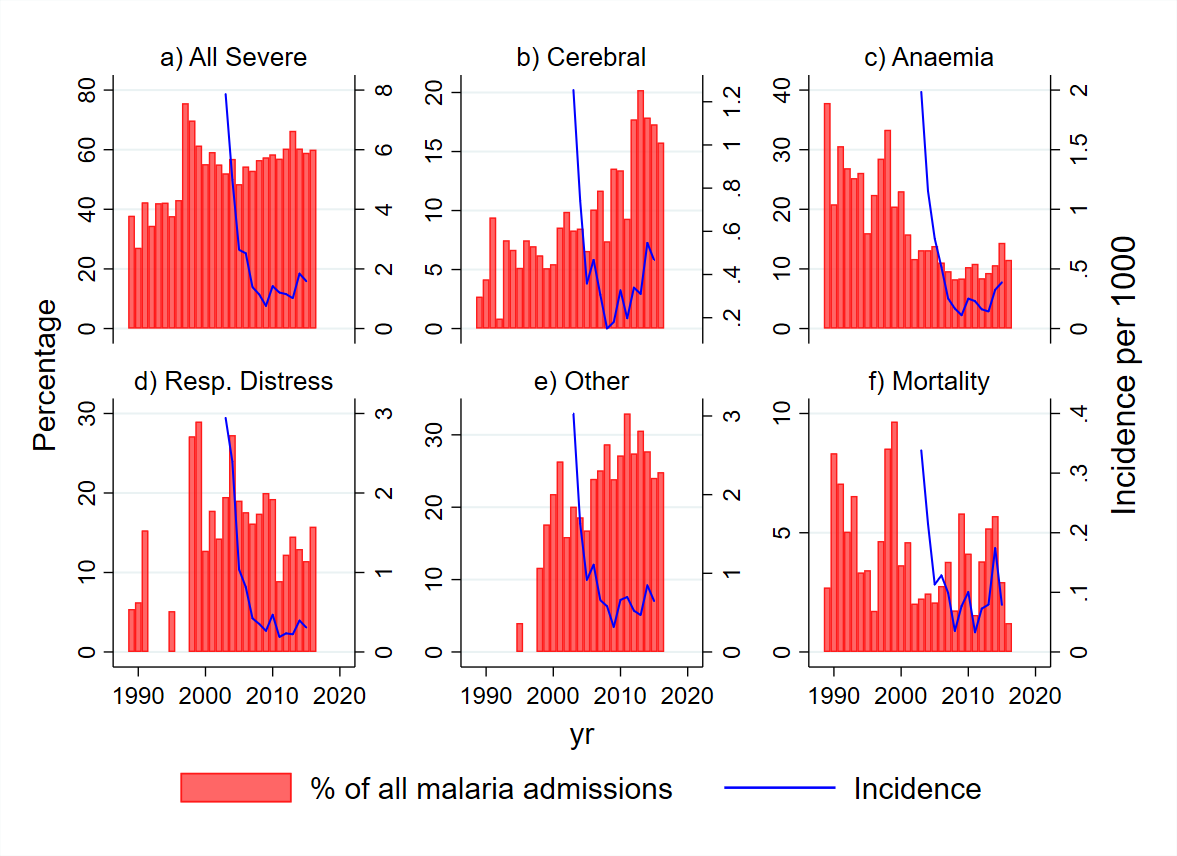

Supplement: Supplementary file 8 — Figure S3. Trends in incidence of mortality and clinical features of severe malaria over time for Kilifi Health and Demographic Surveillance System Residents. The trends over time are shown for clinical features of severe malaria with a parasite threshold of > 2500 (panels a-e) and mortality (panel f); giving the % of all admissions where the relevant observation was positive (red bars, left y axis); and the incidence of cases per 1000 population among under 14 year old children (blue line). (PNG 124 kb) [file 12916_2019_1359_MOESM8_ESM.png]

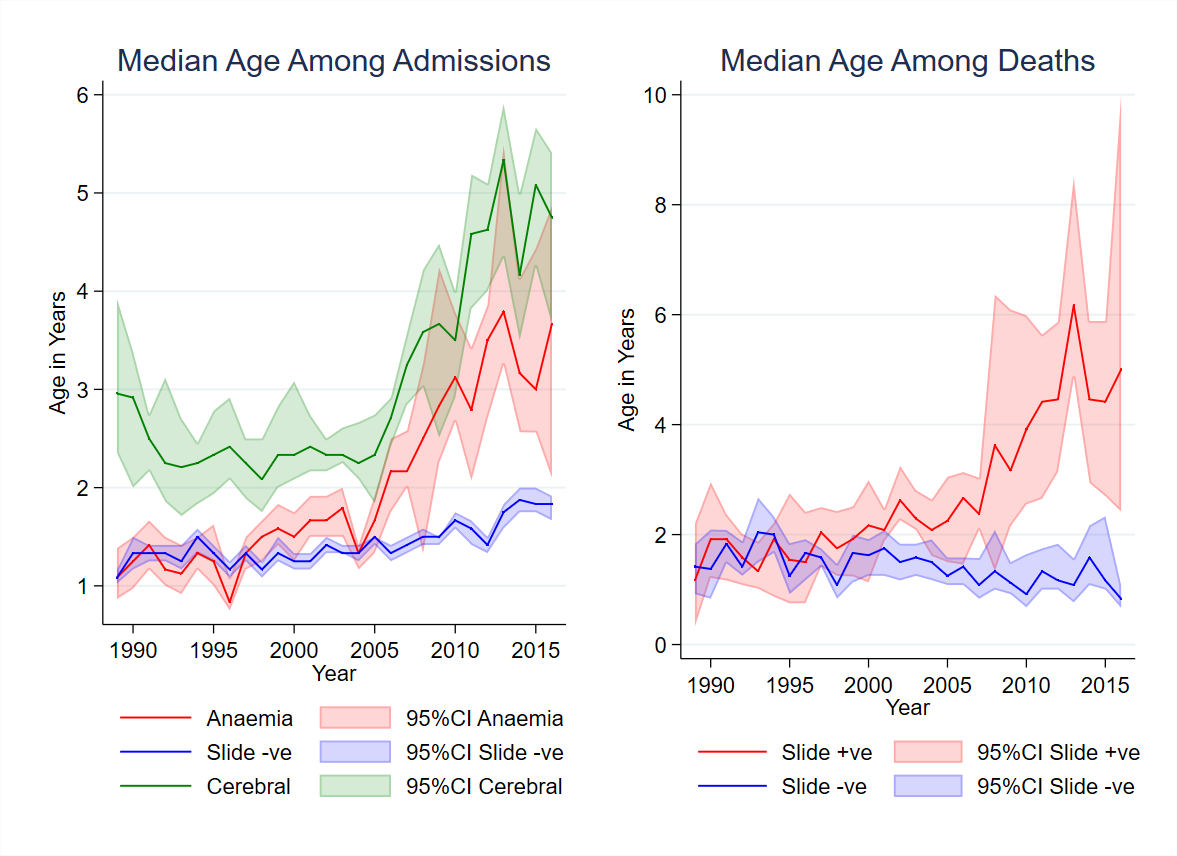

Supplement: Supplementary file 9 — Figure S4. Median ages for children admitted to Kilifi County Hospital. Case definition includes diagnosis by clinician. Median ages of presentation to hospital for specific phenotypes (see color legend) are shown over time with 95% confidence intervals calculated by the binomial exact method. (PNG 152 kb) [file 12916_2019_1359_MOESM9_ESM.png]

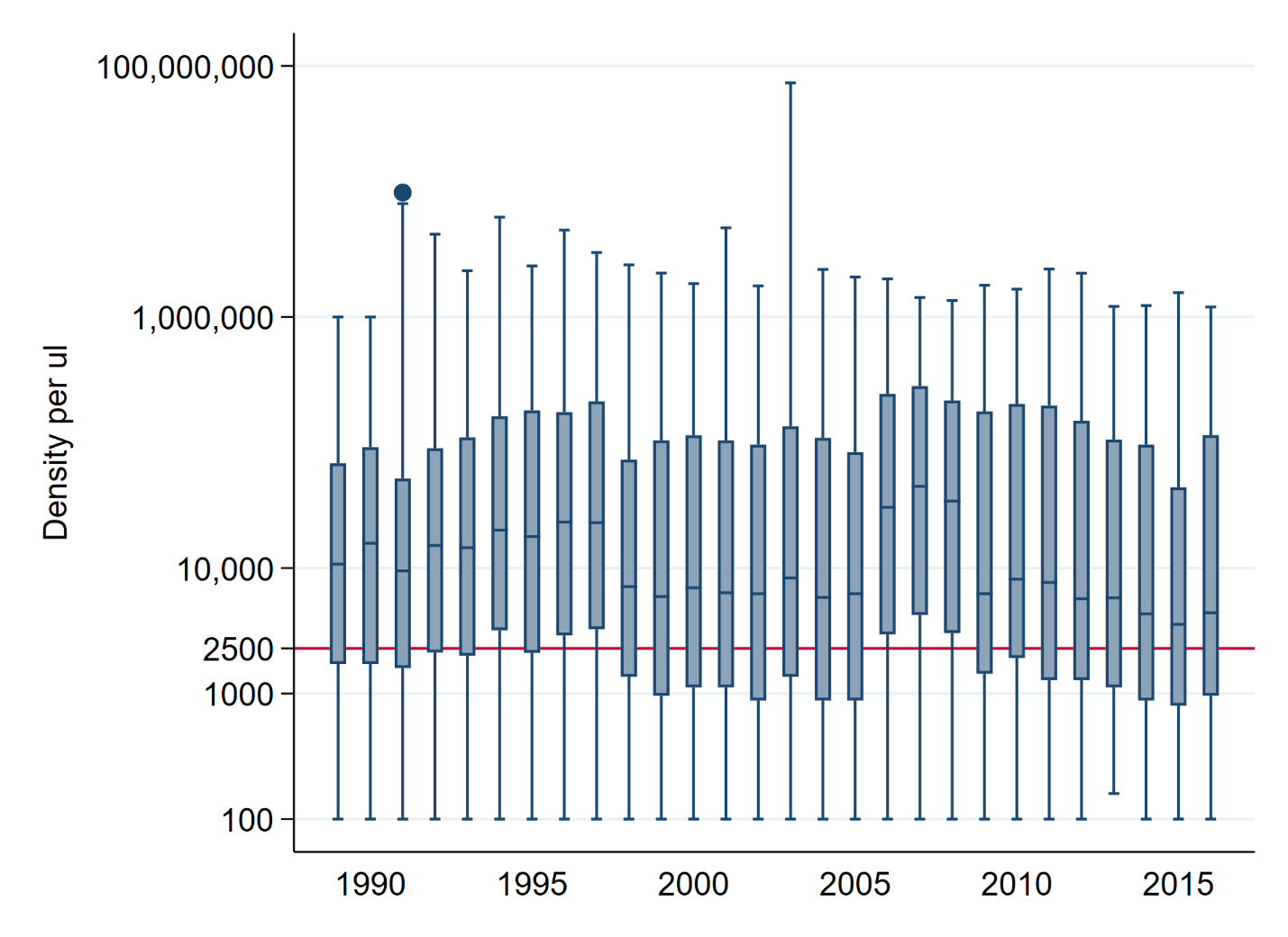

Supplement: Supplementary file 10 — Figure S5. Parasite densities by year. The box plots show median parasite densities, interquartile ranges with boxes and adjacent values (i.e. lowest and highest observations within 1.5 times the interquartile range) with whiskers. Outlying values are shown by circles. (PNG 112 kb) [file 12916_2019_1359_MOESM10_ESM.png]
